# Downregulated genes by silencing *MYC* pathway identified with RNA-SEQ analysis as potential prognostic biomarkers in gastric adenocarcinoma

**DOI:** 10.18632/aging.202260

**Published:** 2020-12-22

**Authors:** Jersey Heitor da Silva Maués, Helem Ferreira Ribeiro, Raquel de Maria Maués Sacramento, Rafael Maia de Sousa, Raíssa Pereira de Tommaso, Bruno Dourado Kovacs Machado Costa, Paulo Cardoso Soares, Paulo Pimentel Assumpção, Caroline de Fátima Aquino Moreira-Nunes, Rommel Mário Rodriguez Burbano

**Affiliations:** 1 Laboratory of Human Cytogenetics, Institute of Biological Sciences, Federal University of Pará, Belém 66075-110, PA, Brazil; 2 Laboratory of Molecular Biology, Ophir Loyola Hospital, Belém, Belém 66063-240, PA, Brazil; 3 Center of Biological and Health Sciences, Department of Biomedicine, University of Amazon, Belém 66060-000, PA, Brazil; 4 Oncology Research Nucleus, University Hospital João de Barros Barreto, Federal University of Pará, Belém 66073-000, PA, Brazil; 5 Laboratory of Pharmacogenetics, Drug Research and Development Center (NPDM), Federal University of Ceará, Fortaleza 60430-275, CE, Brazil

**Keywords:** *MYC*, gastric carcinogenesis, distant metastases, prognosis, biomarkers

## Abstract

*MYC* overexpression is a common phenomenon in gastric carcinogenesis. In this study, we identified genes differentially expressed with a downregulated profile in gastric cancer (GC) cell lines with silenced MYC. The *TTLL12*, *CDKN3*, *CDC16*, *PTPRA*, *MZT2B*, *UBE2T* genes were validated using qRT-PCR, western blot and immunohistochemistry in tissues of 213 patients with diffuse and intestinal GC. We identified high levels of *TTLL12*, *MZT2B*, *CDC16*, *UBE2T*, associated with early and advanced stages, lymph nodes, distant metastases and risk factors such as H. pylori. Our results show that in the diffuse GC the overexpression of *CDC16* and *UBE2T* indicate markers of poor prognosis higher than *TTLL12*. That is, patients with overexpression of these two genes live less than patients with overexpression of *TTLL12*. In the intestinal GC, patients who overexpressed CDC16 had a significantly lower survival rate than patients who overexpressed *MZT2B* and *UBE2T*, indicating in our data a worse prognostic value of *CDC16* compared to the other two genes. *PTPRA* and *CDKN3* proved to be important for assessing tumor progression in the early and advanced stages. In summary, in this study, we identified diagnostic and prognostic biomarkers of GC under the control of *MYC*, related to the cell cycle and the neoplastic process.

## INTRODUCTION

Gastric cancer (GC) is classified globally as one of the most common in the world, with high mortality and a very high incidence, mainly in East Asia, Eastern Europe, and South America [1, 2]. In 2018, data from the GLOBOCAN (Global Cancer Observatory) estimated 1,033,701 new cases of stomach cancer worldwide, representing 5.7% of all new cancer cases. These data also estimated that gastric tumors were the third leading cause of cancer-related death in men and the fifth in women, showing that this disease is more likely to be diagnosed in men than in women [3]. GC is an aggressive disease commonly diagnosed at advanced stages, and surgical resection associated with chemotherapy or chemoradiation is considered the main treatment option [4]. The prognosis of this disease is still poor, partly as a result of local recurrence, tumor invasion, and/or metastasis [5]. The overall relative 5-year survival rate is currently less than 20% [6].

*MYC* dysregulation is a common event in gastric carcinogenesis, including early tumors and premalignant lesions [7]. We have shown in our studies that *MYC* overexpression is an important finding in Brazilian samples [8–12]. Other studies have shown that *MYC* amplification and overexpression was identified in 6-58% of sporadic gastric tumors [13–15], being more frequent in Brazilian samples [7, 16, 17], generally as a result of gene amplification and chromosomal translocations [5, 18]. Although studies show an association of increased *MYC* expression in GC, its function in gastric tumorigenesis is still unclear [19, 20] because most high-performance studies carried out so far on the genetics of GC ignore the importance of MYC in this process [5, 21–25].

In a previous study, we established and characterized three cell lines, AGP01, ACP02 and ACP03, obtained from GC with metastases of an intestinal-type, diffuse-type and intestinal-type, respectively [26]. Those cell lines also carry genetic alterations commonly found in Brazilian GC patients, such as *MYC* amplification and overexpression and *TP53* deletion [7, 27, 28]. Also, these cell lines present the *MYC* silencing that was done through the interference RNA (RNAi), where we explore with bioinformatics tools the transcriptome of these three cell lines to better understand the MYC regulatory signature profile and its targets [29].

In this study, we used an analysis made on public RNA sequencing (RNA-seq) data from two cell lines mentioned above ACP02 – diffuse-type and ACP03 – intestinal-type, both silenced by the expression of *MYC* [29, 30]. Then, we validated the results of the transcriptome using gene expression analysis for three of the top 10 Differentially Expressed Genes (DEGs) of each cell line: *TTLL12*, *CDKN3* and *CDC16* for the ACP02 and *PTPRA*, *MZT2B* and *UBE2T* for the ACP03 in 213 samples of gastric adenocarcinoma and their non-neoplastic pairs, as well as survival data for all patients from the time of diagnosis to the 5-year follow-up. Thus, these genes were chosen among the top 10 most differentially expressed according to the following criteria: be downregulated (regulated positively by MYC), have not yet been described in gastric cancer by our research group and are related to the cell cycle and neoplastic processes pointed out by our bioinformatics analyzes.

## RESULTS

### DEGs after silencing of MYC in ACP02 and ACP03 cell line

We performed an RNA-Seq data analysis from the Gene Expression Omnibus repository (GEO) with accession number GSE81265 to quantify the transcripts and their isoforms in the ACP02 and ACP03 cell lines with the silenced *MYC*, that reduced the expression of this gene by 84% in the ACP02 and 77% in the ACP03. Before silencing, cell phenotypes were heterogeneous, similar to a typical tumor cell line and after silencing, cells became more homogeneous, viable, but significantly lost their tumor capacity for invasion and migration as shown in our previous studies [16, 29].

From a panel of DEGs, we selected only downregulated genes with Log_2_ FC > 1 and *p-value* < 0.01. We use Log_2_ FC [M/C]; where M: *MYC*-siRNA and C: Control-siRNA. We identified 4.098 genes with a downregulated profile in ACP02 and another 842 in ACP03, to be explored in our studies, whose deregulation is directly or indirectly associated with *MYC* amplification, Supplementary Tables 1 and 2.

Thus, six differentially expressed genes (three from ACP02 and three from ACP03) from the top 10 (Table 1) were selected to assess their prognostic and predictive value in clinical specimens of GC tumors that exhibit MYC immunoreactivity. The following genes were selected: *TTLL12* (Tubulin tyrosine ligase-like family member 12), *CDKN3* (Cyclin-dependent kinase inhibitor 3), *CDC16* (Cell division cycle 16), *PTPRA* (Protein tyrosine phosphatase, receptor type, A), *MZT2B* (Mitotic spindle organizing protein 2B) and *UBE2T* (Ubiquitin-conjugating enzyme E2T).

**Table 1:** Top-10 differential expressed genes in gastric cancer.

**The panel of DEGs in a gastric cell line of diffuse histological subtype** **ACP02: MYC-siRNA (2M) vs. Control-siRNA (2C)**								
**Downregulated**					**Upregulated**			
**Gene Symbol**	**Log_2_FC**	***p-value***	**FDR**		**Gene Symbol**	**Log_2_FC**	***p-value***	**FDR**
*UQCRH*	-11.9	4.89E-38	2.70E-37		*ECT2*	12.0	1.47E-58	1.35E-57
*CDC25B*	-11.6	6.73E-18	1.99E-17		*PSMC4*	11.9	2.73E-24	9.98E-24
*CYB561D2*	-11.4	2.30E-51	1.80E-50		*COL8A1*	11.6	2.51E-31	1.15E-30
TTLL12*	-11.4	2.56E-147	1.06E-145		*PSMD11*	11.3	8.94E-34	4.41E-33
*ARHGAP1*	-11.3	3.00E-137	1.10E-135		*BFAR*	11.3	1.97E-26	7.73E-26
CDKN3*	-11.2	3.75E-29	1.61E-28		*YWHAE*	11.3	3.07E-06	5.30E-06
*UBE2B*	-10.9	7.22E-82	1.08E-80		*ABCF1*	11.2	1.97E-26	7.72E-26
*ATP6V0B*	-10.9	2.09E-40	1.23E-39		*PGP*	10.9	1.06E-19	3.33E-19
CDC16*	-10.4	1.38E-49	1.03E-48		*TUSC3*	10.3	1.22E-16	3.42E-16
*UFD1L*	-10.7	1.07E-48	7.79E-48		*CKAP2L*	10.2	1.01E-15	2.73E-15
**The panel of DEGs in a gastric cell line of intestinal histological subtype ACP03: MYC-siRNA (3M) vs. Control-siRNA (3C)**								
**Downregulated**				**Upregulated**			
**Gene Symbol**	**Log_2_FC**	***p-value***	**FDR**		**Gene Symbol**	**Log_2_FC**	***p-value***	**FDR**
PTPRA*	-12.0	2.33E-33	1.56E-31		*COX6A1*	12.3	1.37E-16	1.91E-15
*ATF1*	-11.9	7.33E-30	3.60E-28		*PA2G4*	11.5	1.55E-49	2.66E-47
UBE2T*	-11.3	2.02E-07	8.82E-07		*ATP6V0C*	11.4	4.92E-15	5.77E-14
*CINP*	-10.9	2.97E-06	1.07E-05		*CIB1*	11.3	4.21E-14	4.41E-13
*TERF2*	-10.8	1.87E-16	2.56E-15		*NDUFA7*	11.3	3.39E-08	1.67E-07
*IK*	-10.7	2.47E-10	1.61E-09		*TNFAIP3*	11.2	2.39E-61	6.63E-59
MZT2B*	-10.7	4.34E-05	0.000133		*CDK2AP2*	11.2	9.16E-19	1.64E-17
*RAB23*	-10.6	7.94E-21	1.73E-19		*COX4I1*	11.1	2.26E-10	1.49E-09
*TPD52L2*	-10.5	4.82E-10	3.02E-09		*CCNB1*	10.8	6.11E-21	1.35E-19
*PSMD14*	-10.5	5.29E-08	2.54E-07		*RNF20*	10.8	1.00E-37	9.01E-36

Log_2_ FC [M/C] < 0: Downregulated (left panel); Log_2_ FC [M/C] > 0: Upregulated (right panel); FC: Fold change; FDR: False Discovery Rate.

### Clinical-pathological features and expression of *TTLL12*, *CDKN3*, *CDC16*, *PTPRA*, *MZT2B* and *UBE2T* in gastric cancer

We evaluated the quantitative expression of mRNA, protein and immunohistochemistry of *TTLL12*, *CDKN3*, *CDC16*, *PTPRA*, *MZT2B* and *UBE2T* in 213 patient’s tumor tissues (compared to paired normal gastric tissues) with various clinical and pathological characteristics shown in Supplementary Tables 3 and 4.

*TTLL12* expression was significantly high in the following variables: men under 50 years old, predominantly in tumor tissues located in the cardia, who evolved to a diffuse histological GC, with the early and non-invasive stage (T1/T2), lymph nodes negative (N_0_) and absence of distant metastases.

High levels of *MZT2B* were more significant in women over 50, identified from other parts of the stomach (antrum and body), except for cardia. These patients had a worse evolution in the clinical-pathological variables, indicating gastric cancer of the intestinal-type of serosal invasion in advanced stage (T3/T4). Positive association for lymph nodes, distant metastases and *H. pylori* infection.

We found high levels of *CDC16* and *UBE2T* associated with tumor tissues with intestinal and diffuse GC, respectively. The following common scenarios were observed: predominant in women over 50 years old and with advanced serosal invasion tumors (T3/T4), strongly associated with the presence of lymph nodes and distant metastases.

*CDKN3* and *PTPRA* were expressed in tumor tissue samples from patients of both genders. *CDKN3* was most expressed in tumors of patients over 50 years old, identified mainly in the antrum and the body of the stomach. CDKN3 expression was shown to be associated with the evolution of both types of GC in patients who progressed to a positive serosal invasion of advanced stage (T3/T4), the presence of lymph nodes and distant metastases. *PTPRA* expression was also associated with the evolution of both types of GC in patients aged over 50 years, who have early-stage tumors (T1/T2), with this gene being more expressed in other parts of the stomach than in the cardia.

### Quantification of the gene expression in diffuse and intestinal histological types

**Figure 1:**
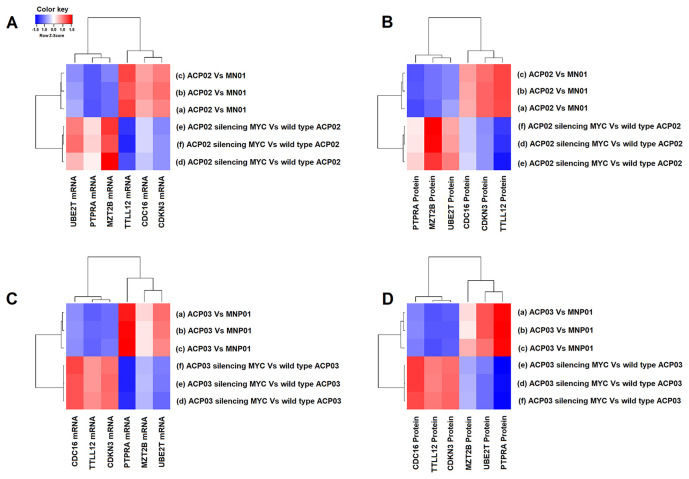
**Effect of MYC silencing on gene and protein expression in ACP02 and ACP03.** The heat maps with hierarchical grouping show how the levels of gene and protein expression of TTLL12, CDKN3, CDC16, PTPRA, MZT2B, UBE2T are related to MYC in the ACP02 (**A**) and (**B**) and ACP03 (**C**) and (**D**). All comparisons were made concerning non-neoplastic gastric mucosa MNP01. Z-score was the metric applied to test the clustering between genes. Blue gradients represent a lower Z-score (genes with a lower level of expression) and red gradients represent a higher Z-score (genes with a higher level of expression).

We quantified the gene expression of the six genes mentioned above in 103 samples of GC tumor tissues of the diffuse-type and 110 of the intestinal-type. The expression data were corroborated by the analysis of protein expression. The increased levels of *MYC* were previously tested in our samples, according to the study by DE SOUZA et al [31]. Figure 1 shows the gene expression results of TTLL12, CDKN3, CDC16, PTPRA, MZT2B, UBE2T generated from the ACP02 and ACP03 cell lines before and after siRNA transfection, Supplementary Table 5.

TTLL12 was more expressed in tumors of diffuse GC [median mRNA (interquartile range, IQR): 1.78 (0.47); median protein (IQR): 1.70 (0.36); *p* <0.001]. In these tumor samples, there was an increase in mRNA and protein by more than 1.5-fold (at least 50% expression) estimated at 86 (83.5%) and 85 (82.5%), respectively (Supplementary Table 3).

*MZT2B* was most expressed in intestinal GC tumor samples [mRNA median (IQR): 1.43 (0.51); protein median (IQR): 1.52 (0.45); *p* <0.001]. Also, mRNA levels reached 48 (43.6%) and the protein increased more than 1.5-fold in 57 (51.8%) in these tumor samples (Supplementary Table 4).

**Figure 2:**
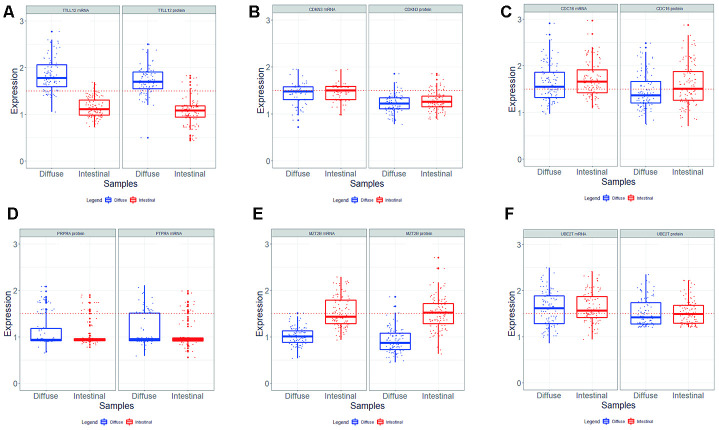
Level of mRNA and protein expression of TTLL12 (**A**), CDKN3 (**B**), CDC16 (**C**), PTPRA (**D**), MZT2B (**E**), and UBE2T (**F**) in diffuse (n = 103) and intestinal (n = 110) GC tumor samples. Mann – Whitney test was used to compare the relative gene expression levels. In all graphs, the expression in gastric tumors was normalized by matched non-neoplastic gastric tissue. RQ: relative quantification; T: tumor sample; N: normal mucosa sample; The whiskers indicate the minimum and maximum values. red dotted line represents the 1.5 fold-change.

We did not find significant differences mRNA and protein expression levels of *CDKN3*, *PTPRA* and *UBE2T* between the two histological types (Supplementary Tables 3 and 4). For *CDC16*, we found only protein levels with a significant increase in intestinal tumor samples (*p* = 0.015). In these tumor samples, we find the following measures of gene expression [mRNA median (IQR): 1.66 (0.51); protein median (IQR): 1.50 (0.63)] (Supplementary Table 3). MRNA and protein levels increased more than 1.5-fold in 77 (70.0%) and 58 (52.7%), respectively. Our results of gene expression are summarized in the graphs of Figure 2.

**Figure 3:**
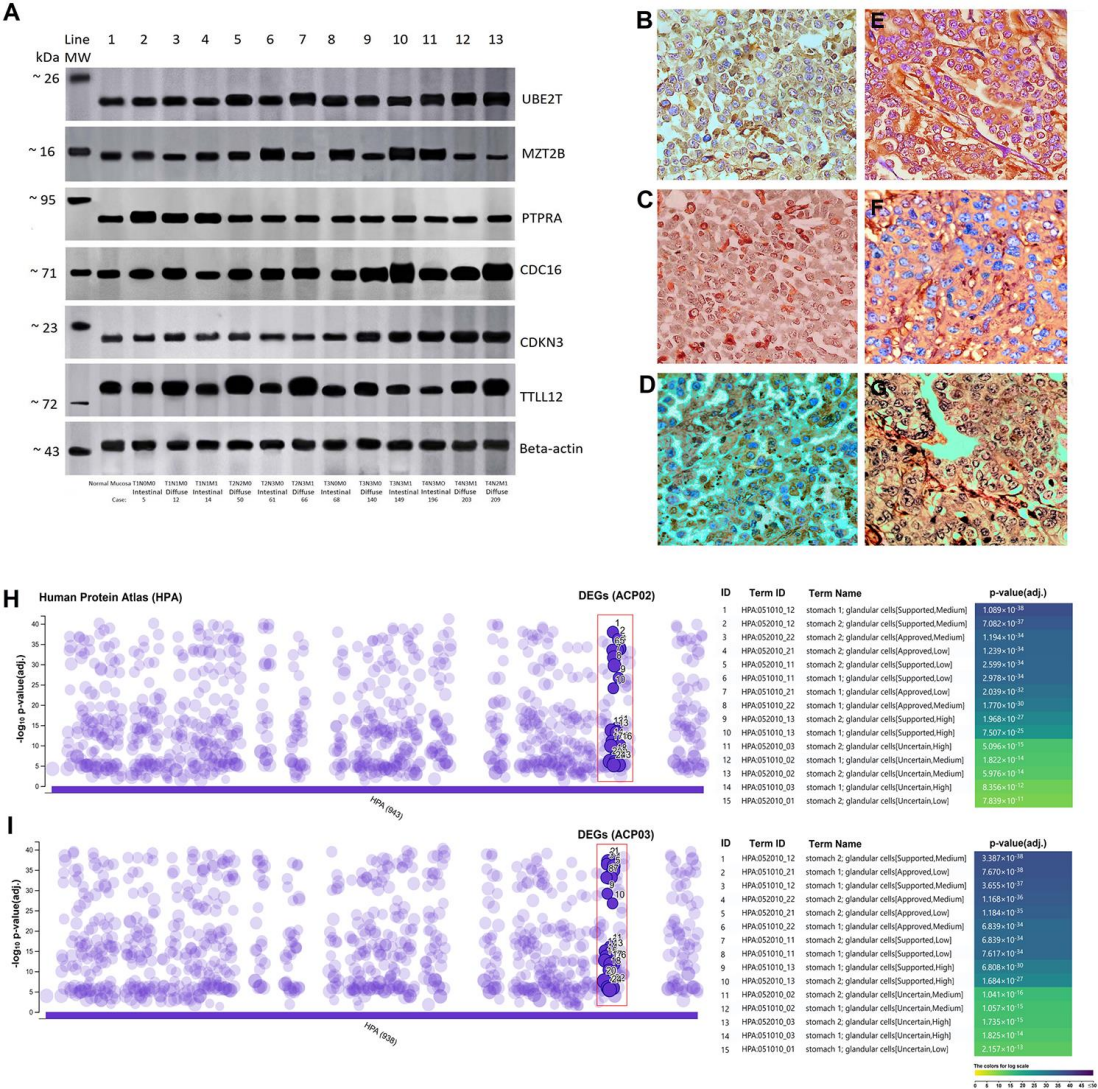
**Analysis of protein expression and immunohistochemistry in gastric cancer subtypes.** (**A**) Representative image of Western-blot. Line 1 represents normal gastric tissue and the intensity of the genes are similar to early TNM stages and without metastasis, but much lower than advanced TNM stages (T3/T4). Initial stages with metastasis also have an intensity much higher than that of normal gastric tissue. (**B**) Positive TTLL12 cytoplasmatic immunostaining in diffuse-type gastric cancer (case 66 T2N3M1); (**C**) Positive CDC16 cytoplasmic and nuclear immunostaining in diffuse-type gastric cancer (case 140 T3N3M0); (**D**) Positive CDKN3 cytoplasmatic immunostaining in diffuse-type gastric cancer (case 203 T4N2M1); (**E**) Positive PTPRA cytoplasmatic immunostaining in intestinal-type gastric cancer (case 5 T1N0M0); (**F**) Positive MZT2B cytoplasmatic immunostaining in intestinal-type gastric cancer (case 61 T2N3M0); (**G**) Positive UBE2T cytoplasmatic and nuclear immunostaining in intestinal-type gastric cancer (case 149 T3N3M1) (magnification x40). The differences in band intensity and intensity of immunoreactivity are due to the different stages of TNM in tumor samples of diffuse and intestinal histological types that represent figures (3A, 3B–3G). (**H** and **I**) The function of the DEGs in the gastric lines showed a strong correlation between the increased level of protein expression with the human stomach cells in the data from The Human Protein Atlas (HPA) that were accessed and normalized in -log_10_ *p*-value (adj.). The levels of protein expression of the DEGs are identified by the red rectangle in 15 subtypes of human stomach cells.

Corroborating the results of qPCR, western blot and immunoreactivity, it can reveal significant differences of expression in samples with different TNM and in different histological types (Figures 3A and 3B–3G). Also, our protein expression results for the DEGs of the ACP02 and ACP03 were shown to be correlated with those of The Human Protein Atlas, revealing significant expression profiles in the stomach cells (Figure 3H–3I). Relationship between the expression of *TTLL12*, *MZT2B*, *CDC16* and *UBE2T* with metastatic progression.

**Figure 4:**
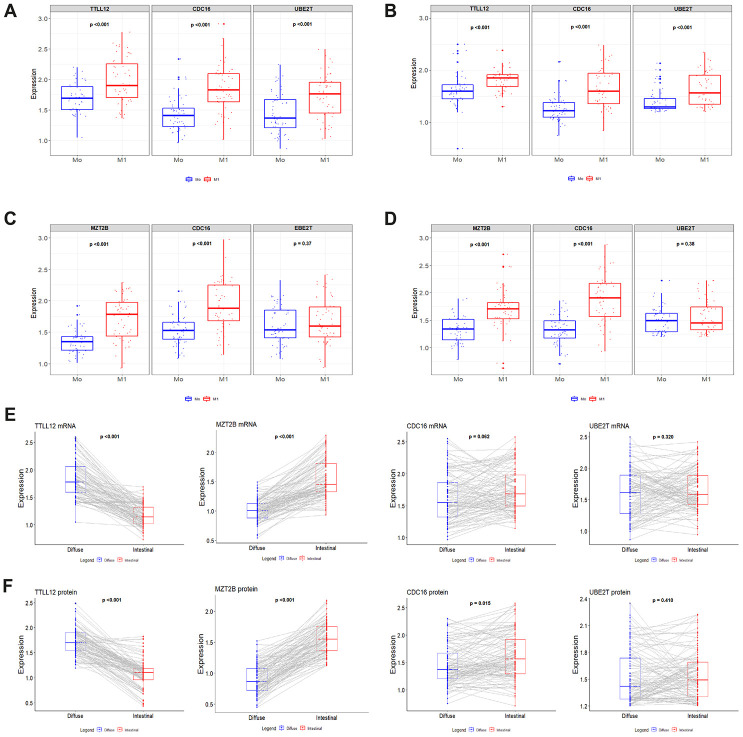
**Box plot shows the relationship between the normalized expression of *TTLL12*, *MZT2B*, *CDC16* and *UBE2T* with metastatic progression.** (**A** and **B**) mRNA and protein levels of *TTLL12*, *CDC16* and *UBE2T* in 103 tissue samples with diffuse-type GC from patients M_0_ and M_1_. (**C** and **D**) mRNA and protein of *MZT2B*, *CDC16* and *UBE2T* in 110 tissue samples with intestinal-type GC from patients M_0_ and M_1_. The boxes are drawn from the 75^th^ to the 25^th^ percentile. The vertical lines above and below the box define the maximum and minimum values and the dots indicate outliers, the horizontal line inside the box representing the median. (**E** and **F**) mRNA and protein of *TTLL12*, *MZT2B*, *CDC16* and *UBE2T* in patients with diffuse (n = 103) and intestinal (n = 110) GC that were associated with M_0_ and M_1_. The lines with the gray gradient represent the connections of points M_0_ and M_1_ that vary according to the normalized expression value. The expression levels of these genes were validated by qRT-PCR and western blot in 213 patients. (M) presence of metastasis. Mann – Whitney test was used to compare the relative gene expression levels.

Gene expression results from Supplementary Tables 3 and 4 was used to establish an association between the increase in mRNA and protein of the *TTLL12*, *MZT2B*, *CDC16* and *UBE2T* genes with a possible metastatic progression of patients M_0_ and M_1_. We identified increased levels of *TTLL12*, *CDC16* and *UBE2T* in tumor samples from 103 patients with diffuse GC (M_1_) (*p* <0.001 for all analyzes; Figure 4A and 4B).

Likewise, we identified an increase in mRNA and protein levels in tumor samples from 110 patients with intestinal GC (M_1_), for *MZT2B* and *CDC16* (*p* <0.001 for all analyzes; Figure 4C and 4D). These results are corroborated when we evaluate the increased levels of mRNA and protein in tumor samples from patients M_1_ that were analyzed by histological types (Figure 4E and 4F). These results differ from those found in Supplementary Tables 3 and 4 because it takes into account the total samples (n = 213).

### Association of the expression profile with survival

**Figure 5:**
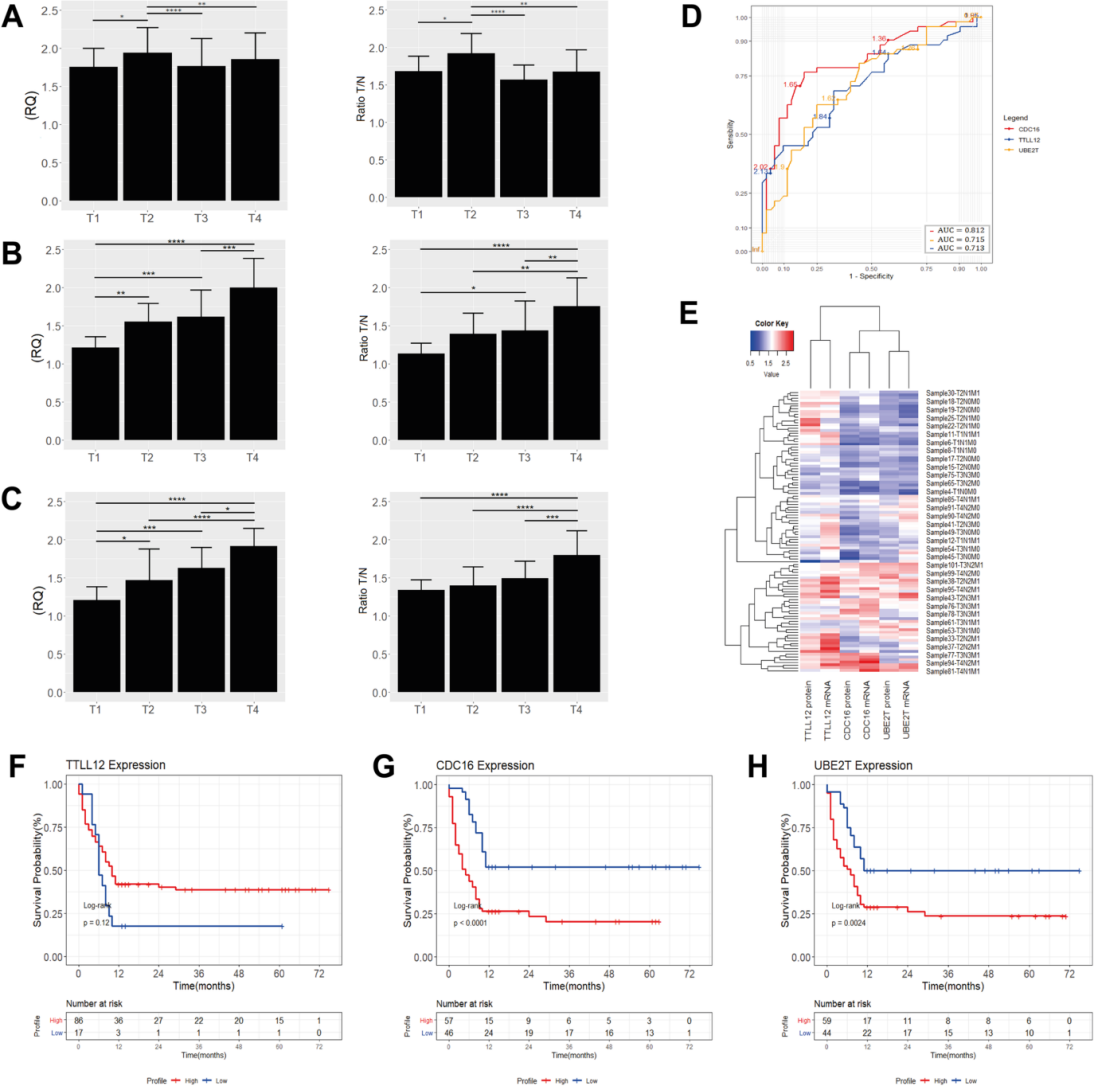
Quantification of *TTLL12* (**A**), *CDC16* (**B**) and *UBE2T* (**C**) mRNA and protein levels by tumor stage in GC of the diffuse-type. *p <0.008, **p <0.001, ***p <0.0002, ****p <0.0001, significant difference between groups (Nonparametric Mann – Whitney test). In all graphs, the expression in gastric tumors was normalized by matched non-neoplastic gastric tissue. RQ: relative quantification; T: tumor sample; N: normal mucosa sample. (**D**) ROC curve analysis. The cutoff point was chosen as the highest point of the Area-Under-the-Curve (AUC) for *TTLL12*, AUC: 0.713; (95% CI 0.62-0.76) with 76.02% sensitivity and 50.04% specificity, for *CDC16*, AUC: 0.812; (95% CI 0.70-0.83) with 85.0% sensitivity and 50.08% specificity and for *UBE2T*, AUC: 0.715; (95% CI 0.63-0.77) with 77.06% sensitivity and 48.03% specificity. (**E**) Heat maps showing the average levels of gene expression of ~35 samples of GC tissues of the diffuse-type. The red gradient shows the highest levels of expression while the blue gradient shows the lowest levels. Kaplan-Meier analysis of the overall survival (in months) of patients with diffuse gastric cancer as a function of (**F**) *TTLL12*, (**G**) *CDC16* and (**H**) *UBE2T* expression. We analyzed high expression (gene expression ≥1.5; red line), as opposed to low expression (gene expression <1.5; blue line) associated with a higher probability of survival. We found more significant values when we associated the high expression of *CDC16* (*p* <0.0001) and *UBE2T* (*p* = 0.0024), with patients who had a shorter survival.

**Figure 6:**
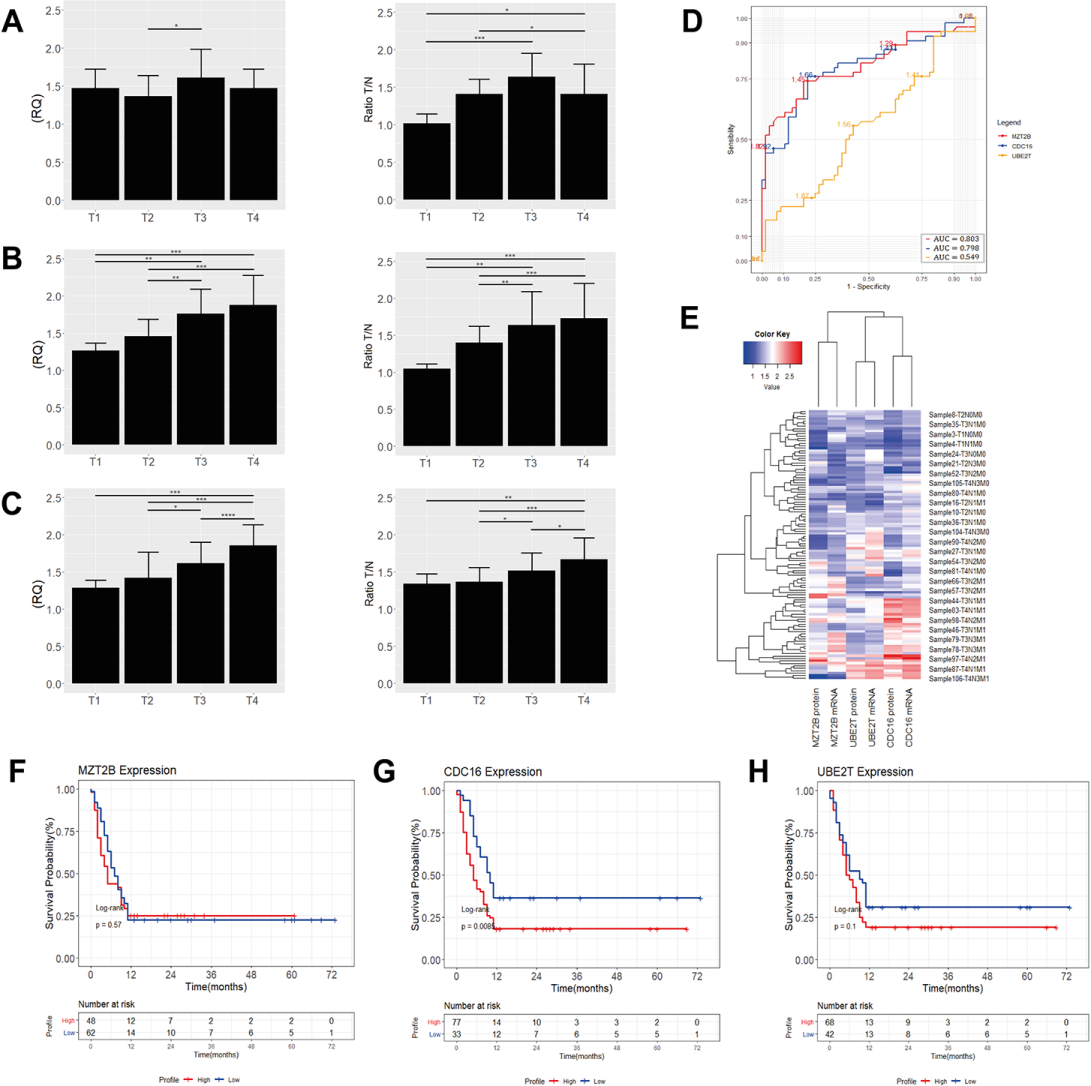
Quantification of *MZT2B* (**A**), *CDC16* (**B**) and *UBE2T* (**C**) mRNA and protein levels by tumor stage in GC of the diffuse intestinal. *p <0.008, **p <0.001, ***p <0.0002, ****p <0.0001 significant difference between groups (Nonparametric Mann – Whitney test). In all graphs, the expression in gastric tumors was normalized by matched non-neoplastic gastric tissue. RQ: relative quantification; T: tumor sample; N: normal mucosa sample. (**D**) ROC curve analysis. The cutoff point was chosen as the highest point of the Area-Under-the-Curve (AUC) for *MZT2B*, AUC: 0.803; (95% CI 0.69-0.82) with 84.2% sensitivity and 50.0% specificity, for *CDC16*, AUC: 0.798; (95% CI 0.69-0.82) with 86.0% sensitivity and 49.08% specificity and for *UBE2T*, AUC: 0.549; (95% CI 0.51-0.68) with 52.03% sensitivity and 47.05% specificity. (**E**) Heat maps showing the average levels of gene expression of ~30 samples of GC tissues of the intestinal-type. The red gradient shows the highest levels of expression while the blue gradient shows the lowest levels. Kaplan-Meier analysis of the overall survival (in months) of patients with gastric cancer of the intestinal-type as a function of the expression of (**F**) *MZT2B*, (**G**) *CDC16* and (**H**) *UBE2T*. We analyzed high expression (gene expression ≥1.45; red line), as opposed to low expression (gene expression <1.45; blue line) associated with a higher probability of survival. We found more significant values only for high expression of *CDC16* (*p* <0.0085) when associated with the lower shortest patient survival.

Our results of TTLL12, MZT2B, CDC16 and UBE2T expressions showed that higher levels of TTLL12 are associated to early-stage tumors (T1/T2) in the diffuse-type (Figure 5A). in early-stage tumors (T1/T2) (Figure 5A). We identified higher levels of *CDC16* and *UBE2T* expression in diffuse advanced-stage GC tumors (T3/T4) (Figure 5B, 5C). InGCtumors of the intestinal-type, the expressions of *MZT2B*, *CDC16* and *UBE2T* were higher only in advanced-stage tumors (T3/T4) (Figure 6A–6C).

Analysis of the area under the ROC curve (AUC) to classify patients with high and low gene expression associated with survival capacity showed a cutoff point as the highest point of the AUC for the *CDC16*: AUC = 0.812, *p* <0.0001; followed by *TTLL12* and *UBE2T* that exhibited practically the same area under the curve with AUC = 0.713, *p* <0.0001 and AUC = 0.715, *p* <0.0001, respectively (Figure 5D). We identified the cut-off values of 1.65 for *CDC16*, 1.64 for *TTLL12* and 1.56 for *UBE2T*. The same previous analysis indicated for *MZT2B*: AUC = 0.803, *p* <0.0001; *CDC16*: AUC = 0.798, *p* <0.0001; *UBE2T*: AUC = 0.549, *p* <0.01. From these data, we identified cut-off values of 1.45 for *MZT2B*, 1.65 for *CDC16* and 1.56 for *UBE2T* (Figure 6D). The heat maps show the levels of gene expression in ~ 35 samples of GC tissues from patients M_0_ and M_1_ of the diffuse and ~ 30 of the intestinal, confirming how the gene expression increases according to the tumor stage (Figures 5E and 6E).

In the first year after the diagnosis of diffuse GC, patients with high expression of *TTLL12* (Log-rank test, *p* = 0.12) showed no association with decreased survival and their overexpression is not related to poor prognosis, as patients with higher levels of *TTLL12* exhibited increased survival. On the other hand, the Kaplan-Meier analysis demonstrated an association between the high expression of the *CDC16* (Log-rank test, *p* <0.0001) and *UBE2T* (Log-rank test, *p* = 0.0024) with decreased overall survival patients, indicating a worse prognosis (Figure 5F–5H).

In the first year after the diagnosis of intestinal GC, the Kaplan-Meier analysis showed a significant result only for the association between the high expression of *CDC16* (Log-rank test, *p* = 0.0085) with the decrease in the overall survival of the patients and consequently, worse prognosis. Patients with high expression of *MZT2B* (Log-rank test, *p* = 0.57) and *UBE2T* (Log-rank test, *p* = 0.100) did not show a significant association with decreased survival (Figure 6F–6H).

### Gene set enrichment analysis (GSEA) in ACP02 and ACP03

**Figure 7:**
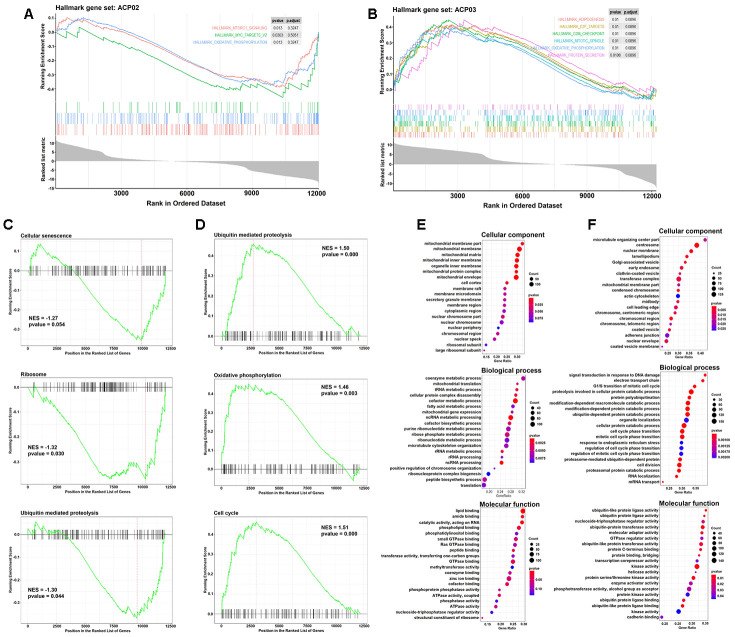
**GSEA analysis of the DEGs in ACP02 and ACP03.** (**A** and **B**) GSEA Enrichment plot of the main sets of genes enriched in the hallmark collection in both cell lines. Three major important KEGG pathways were inhibited in ACP02 (**C**) and activated in ACP03 (**D**). GO (Gene ontology) analysis was identified in the transcriptomes in ACP02 (**E**) and ACP03 (**F**) for the categories of cellular component, biological process, and molecular function. In the hallmark and KEGG charts, the enrichment score values assume negative values (rank of inhibited genes) for ACP02 and positive values (rank for activated genes) for ACP03, as indicated in the curves. The FDR is the estimated probability that a gene set with a given NES (normalized enrichment score) represents a false-positive finding. Several enriched gene sets are significant as indicated by an FDR of less than 25%. In all analyzes *p-*value <5%.

We identified increased and decreased pathways in both gastric cells line, based on hallmark gene sets (“Hallmark gene sets summarize and represent specific well-defined biological states or processes and display coherent expression”, as defined by GSEA), KEGG and gene ontology (Figure 7).

In the ACP02 we identified a decrease in the expression of genes related to the hallmark gene set that was more significant for mTORC1 signaling, MYC targets and Oxidative phosphorylation, while in the ACP03 we identified an increase in the expression of more significant genes for Adipogenesis, E2F targets, G2M checkpoint, mitotic spindle, oxidative phosphorylation and protein secretion (Figure 7A, 7B).

We still found a wide repertoire of KEGG metabolic pathways inhibited in ACP02, mainly for cellular senescence, ribosome, ubiquitin-mediated proteolysis (Figure 7C). The analysis of the ontology gene revealed many genes of this lineage related to a mitochondrial cell component, playing a role in the metabolic process of lipids, phospholipids, amino acids and RNA catalytic activities, in addition to several molecular functions related to small GTPases (Figure 7E).

The main pathways activated in ACP03 were for ubiquitin-mediated proteolysis, oxidative phosphorylation, cell cycle (Figure 7D). Most of the genes activated in these cells were noted, mainly, in nuclear components and organelles such as the Golgi apparatus and mitochondria, exercising functions in protein catabolism, signal transduction and cell cycle (Figure 7F). The total result of this analysis can be accessed in Supplementary Table 6.

## DISCUSSION

*MYC* has a fundamental role in gastric carcinogenesis, being one of the most robust and significant prognostic markers in the GC [32]. *MYC* amplification and its upregulated expression were commonly observed in cell lines and tissues of GC. The highest *MYC* levels have been reported in the tumors of patients with local or distant metastasis [7, 8, 11, 12, 28, 31–36]. It is therefore critical to understand how the *MYC* knockdown alters the expression of several genes involved in gastric carcinogenesis, being an important step in the discovery of new targets activated by this gene [37, 38].

In this work, qPCR and western blot results revealed a significant increase in expression levels of *TTLL12*, *MZT2B*, *CDC16* and *UBE2T* in GC tumor samples of diffuse and intestinal-type, when compared with normal gastric tissues. We also evaluated the levels of CDKN3 and PTPRA that were not efficient to differentiate the two histological types of the disease. They are useful only for identifying the GC.

The expression of *TTLL12* was more significant in the GC of the diffuse-type located in the cardia region of male patients under 50 years of age, who had the disease at an early-stage and a longer survival during the first year after diagnosis, as found in other studies [39, 40]. Such characteristics added to a high prevalence of this neoplasia in the north and northeast of Brazil [41], reinforces the importance of an early screening method for this type of GC. This gene can be used as a biomarker to identify diffuse GC early, as we have identified increased levels of *TTLL12* in tumor samples from patients predisposed to metastatic progression. *TTLL12* is a gene capable of modifying tubulins, a marker that can be studied and deepened in the progression and metastasis of GC. Studies show that the levels of the c-MYC oncoprotein activated *in vitro* and *in vivo* conditions exert its effects on alpha-tubulin and polymerized microtubules [42, 43]. TTLL12 is reported to be abnormal in many cancer cells [44–46], but its function in GC is still unknown.

The high expression of MZT2B was more significant in GC samples of the intestinal histological type, identified exclusively in the antrum and the stomach body [47] of female patients over 50 years of age. These patients had worse clinical and pathological evolution, developing distant metastases, infection by *H. pylori* and shorter survival. To date, only one study has identified *MZT2B* as a target for MYC in gene expression data for breast cancer cell lines and tissues [48]. MZT2B is an important binding protein in the organization of the mitotic spindle. We suggest that the elevated expression of MZT2B is activated by MYC in gastric carcinogenesis resulting in dramatic changes in the extracellular matrix and the cell communication pathways [49] associated with tumor progression, intestinal metaplasia and exposure to risk factors such as *H. pylori* infection [50].

*CDC16* and *UBE2T* showed high levels in the intestinal and diffuse GC, capable of compromising the antrum region and the stomach body, especially in women over 50 years old. The increased expression of these two genes in the diffuse GC is associated with decreased survival, related to the variables of worse prognosis, which was not observed in the survival results of patients who expressed UBE2T in the intestinal-type. MYC can activate the protein encoded by CDC16 which functions as an anaphase promoter complex, mediated by a ubiquitin ligase that activates the progression of mitosis and the G1 phase of the cell cycle, controlling the onset of DNA replication [51].

Studies with CDC16 in the GC are still scarce, but the involvement of this gene has been reported in metastasis of prostate [52], lung [53] and human colon [54]. The function of UBE2T has been reported in the progression of GC as a potential prognostic biomarker [55]. The ubiquitin-conjugating enzyme E2T (UBE2T) is a member of the E2 family that mediates the ubiquitin-proteasome system that acts on gene expression [55, 56]. UBE2T activates cell mobility, inducing the fundamental epithelial-mesenchymal transition in gastric tumorigenesis.

The expression levels of *CDKN3* and *PTPRA* were quantified without significant differences between the two histological types, representing two important biomarkers to detect GC without differentiating the disease. CDKN3 was more associated with the worst prognosis variables in the advanced GC than PTPRA, which was more associated with the variables with a better prognosis. CDKN3 cyclin-dependent kinase inhibitor 3, is a cyclin-dependent kinase (CDKs) that controls cell cycle progression and other critical functions within the cell [57]. *CDKN3* can be recruited by MYC to keep many mitotic cells responsible for promoting tumorigenesis [58]. MYC can activate tyrosine phosphatase signaling pathways which are important mechanisms involved in signal transduction in cancers [59]. Possibly, MYC increases the levels of PTPRA, activating the function of this gene as a tumor suppressor, enabling cell signaling events, coordinating the control of proliferation, apoptosis, survival, migration and invasion [60, 61].

Our results of functional annotation of the DEGs of the gastric cell lines revealed a strong correlation between the increase in protein expression with the stomach cells. Confirming the effects of MYC on the gene expression of these cell lines. We still found strong evidence that the silencing of MYC in both cells’ lines, activated and inhibited some sets of genes and important carcinogenesis pathways with a direct impact on cell senescence, ribosome assembly, ubiquitin-mediated by proteolysis, oxidative phosphorylation, cell cycle control, strongly associated with *TTLL12*, *CDKN3*, *CDC16*, *PTPRA*, *MZT2B* and *UBE2T*.

The identification of new diagnostic biomarkers and prognoses of the GC is urgently needed. Approaches involving integrated bioinformatics for mining transcriptomic, epigenomic and other types of broad genome associations are effective tools for identifying new biomarkers associated with pathways and modules related to disease subtypes [62–64]. We suggest that only the analysis of MYC-activated genes in cell lines is not entirely conclusive. The most important information comes from validating data collected from clinical samples of tumors and surrounding normal tissues. The genes identified in this study proved to be promising biomarkers for the identification of GC and have been validated in a considerable number of samples (210). However, to consolidate their clinical applicability, we strongly recommend that they be included in evaluations on large scales of patients (above 1000).

## CONCLUSION

In summary, our results showed that in the diffuse GC, overexpression of *CDC16* and *UBE2T* indicate markers of poor prognosis higher than *TTLL12*, that is, patients with overexpression of these two genes live less than patients with overexpression of *TTLL12*. In the intestinal GC, patients who overexpressed *CDC16* had significantly shorter survival than patients who overexpressed *MZT2B* and *UBE2T*, confirming the worse prognostic value of *CDC16* in this disease. We also emphasize the importance of *PTPRA* and *CDKN3*, which are promising to assess tumor progression in early and advanced stages of the GC, without differentiating the histological types.

## MATERIALS AND METHODS

### Cell lines used in this study

In this study, ACP02 and ACP03 cell lines that were obtained from diffuse and intestinal GC, respectively established and previously characterized by our group, were used [26]. Cell culture of non-neoplastic gastric mucosa cells (Normal Gastric Mucosal Cell Line 01, MNP01) was also used to initially evaluate the gene and protein expression of *TTLL12*, *CDKN3*, *CDC16*, *PTPRA*, *MZT2B* and *UBE2T*. The ACP02, ACP03 and MNP01 cell lines were cultured in Dulbecco’s modified Eagle’s medium (DMEM; Gibco/Invitrogen, Germany) supplemented with 10% fetal bovine serum (Gibco/Invitrogen, Germany), 100 U/ml penicillin, 100 μg/ml streptomycin, and 0.25 μg/ml amphotericin B. All cultures were maintained in a 5% CO_2_ air-humidified atmosphere at 37° C [16].

### *MYC*-silencing in ACP02 and ACP03

A total of 2x10^5^ ACP02 and ACP03 cells were seeded in 12-well culture plates after 24 h *MYC* silencing was done by transfecting ACP02 and ACP03 cells with a solution of Lipofectamine® RNAiMAX Transfect Reagent (Life Technologies, USA) diluted in Opti. -MEM® (Life Technologies, USA) mixed with the control siRNA or the target gene-targeted siRNA. SiRNAs used against the *MYC* gene constituted a pool of four different commercially obtained previously validated synthetic siRNAs (SMARTpool ON-TARGETplus MYC siRNA L-003282-02-0020, Dharmacon, GE Lifesciences, USA) at a concentration of 20 μM; The negative control was a 4 siRNA pool whose sequence was not the target of any known transcript (ON-TARGETplus Non-targeting Pool D-001810-10-05, Dharmacon, GE Lifesciences, USA).

For *MYC* silencing, a pool of four different double-stranded siRNAs targeting MYC (20 μM; SMARTpool ON-TARGETplus MYC siRNA, L-003282-02-0020; GE Healthcare Dharmacon, USA) or scrambled control siRNAs (ON-TARGETplus Nontargeting Pool, D-001810-10-05; GE Healthcare Dharmacon, USA) were transfected into ACP02 and ACP03 cell lines using Lipofectamine RNAiMAX Transfection Reagent (Life Technologies, USA). All siRNA experiments were performed three times.

### Data analysis

In this study, we used public RNA-Seq data that was obtained with Ion Proton™ from two gastric cancer cell lines ACP02 and ACP03. This data is deposited in the Gene Expression Omnibus (GEO) under the access number GSE81265, which was previously analyzed [29]. In our analysis, ACP02 cell line control was named 2C, and its *MYC*-silenced counterpart was named 2M. Similarly, ACP03 cell line control was termed as 3C and the *MYC*-silenced was named 3M.

### Identification and analysis of DEGs

Differential Expressed Genes (DEGs) were identified between *MYC*-siRNA (M) and control-siRNA (C) from ACP02 and ACP03 lines. We use the following comparisons in the ACP02 cell line (2M versus 2C) and the ACP03 cell line (3M versus 3C). To identify the DEGs between two paired samples, we used the Audic-Claverie test [65]. DEGs with a cutoff point of [| Log_2_ Fold change (FC) | > 1) and *p*-value <0.01, FDR <0.01] were calculated as the Log_2_ ratio between the silenced and the control sample. Among the top-10 downregulated genes evaluated in the silenced *MYC* cell lines, six genes were selected (*TTLL12*, *CDKN3*, *CDC16*, *PTPRA*, *MZT2B* and *UBE2T*) and validated by qPCR, western blot and immunohistochemistry in samples of tumor tissues of patients with GC. Such genes were selected because we consulted several studies in the literature reporting their involvement with other types of cancer, but we did not find a direct implication with the GC.

### Functional analysis of DEGs in gastric cancer

The functional analysis of the DEGs was done in two moments. First, we used the g: Profiler [66] tool to investigate the correlation between the protein expression of the ACP02 and ACP03 DEGs with stomach cells using The Human Protein Atlas (https://www.proteinatlas.org/). Protein expression data were normalized on the -log_10_ p-value scale (adj.), which showed significance for 15 different cell types of the human stomach.

Then, the DEGs of the ACP02 and ACP03 lines were analyzed separately with the fgsea package [67]. Complementary analyzes were done with ClusterProfiler packages [68]. We used three collections of gene expression data from The Molecular Signatures Database (MSigDB, https://www.gsea-msigdb.org/gsea/msigdb), Hallmark gene set, Gene Ontology and KEGG: Kyoto Encyclopedia of Genes and Genomes. Permutation was conducted 1,000 times according to default-weighted enrichment statistics and by using a Signal2Noise metric to rank genes. Set genes with 10 to 500 genes and FDR <25% were selected for evaluation. Significant gene sets were defined as those with a nominal *p*-value <0.05.

### Patients and tissue specimens

A total of 213 patients’ samples of gastric adenocarcinoma and its non-neoplastic counterparts were obtained with local Ethics Committee approval number: 2.340.667 from the Ophir Loyola Hospital, Belém, Brazil. Signed informed consent was obtained from all patients before sample collection. All methods were carried out following the relevant guidelines and regulations. All samples were classified according to Lauren and the tumors were staged according to the tumor-node-metastasis (TNM) staging criteria [69]. The presence of *Helicobacter pylori* (*H. pylori*) in gastric samples was detected by the rapid urease test, and its virulence factor cytotoxicity-associated gene A (CagA gene) was detected by polymerase chain reaction (PCR) using DNA purified simultaneously with proteins and mRNA, as previously performed by our group [70]. *Epstein-Barr virus* (EBV) was detected by RNA *in situ* hybridization.

### mRNA expression

After the total mRNA was isolated, it was reverse-transcribed using the High-Capacity cDNA kit according to the manufacturer’s protocol (Thermo Fisher Scientific, USA). The mRNA and cDNA concentration and quality were determined using a NanoDrop spectrophotometer (Kisker, Germany) and 1% agarose gels, respectively. Samples were stored at -80 ° C until use. cDNA was then amplified by real-time quantitative PCR (qPCR) using TaqMan probes: *TTLL12*: Hs00209450_m1, *CDKN3*: Hs00193192_m1, *CDC16*: Hs00187430_m1, *PTPRA*: Hs00160751_m1, *MZT2B*: Hs01117110_sH, *UBE2T*: Hs00928040_m1 and *ACTB* (4333762F; Thermo Fisher Scientific, USA) gene was selected as an internal control [27]. All qPCRs were performed in triplicate in 7500 Fast Real-Time PCR instrument (Thermo Fisher Scientific, USA).

The relative quantification of gene expression was calculated according to the method of Livak and Schmittgen [71]. We used a control sample of non-neoplastic gastric mucosa cells MNP01 (Normal gastric mucosa cell Line 01) pooled from 10 healthy patients [16], which was used as a calibrator for each tumoral sample. The mRNA and protein data are expressed as the median and interquartile range (IQR) of fold change in gene expression level in the gastric tumors normalized to the *ACTB* gene and relative to levels in the adjacent non-neoplastic control sample.

### Protein quantification

Western blot analysis was performed as described previously [16]. Reduced protein (25 μg) from each sample was applied to SDS-polyacrylamide gel and electrophoresed. Then the individual proteins in the electrophoresis gel were transferred to a polyvinylidene fluoride membrane and labeled with antibodies specific for the corresponding DEG proteins: anti-TTLL12 (dilution 1:2000; MA5-25018; Thermo Fisher Scientific, USA) anti-CDKN3 (1:2000 dilution; MA5-25690; Thermo Fisher Scientific, USA), anti-CDC16 (1:1000 dilution; MA5-32406; Thermo Fisher Scientific, USA), anti-PTPRA (dilution; 1:1000; PA5-15522; Thermo Fisher Scientific, USA), anti-MZT2B (Santa Cruz Biotechnology, USA; dilution 1:1000 sc-514516), anti-UBE2T (1:500; MA5-25226; Thermo Fisher Scientific, USA) and ACTB (dilution 1:250; Ac-15), ACTB was used as a loading reference control.

### Immunohistochemistry

Immunoreactivity of proteins encoded by the selected genes *TTLL12* (1:50 dilution; MA5-25018; Thermo Fisher Scientific, USA), *CDKN3* (dilution 1:150; MA5-25690; Thermo Fisher Scientific, USA), *CDC16* (dilution 1:150; MA5-32406; Thermo Fisher Scientific, USA), *PTPRA* (dilution; 1: 100; PA5-15522; Thermo Fisher Scientific, USA), *MZT2B* (Santa Cruz Biotechnology, USA; 1:1000 dilution sc-514516), *UBE2T* (1:50; MA5-25226; Thermo Fisher Scientific, USA) was evaluated by immunohistochemistry on paraffin-embedded tissue sections, as suggested by Calcagno et al. [11].

### Statistical analysis

The validation data are shown as the frequency, median, and interquartile range (IQR). The Mann-Whitney test was used to investigate the possible associations between gene mRNA or protein expression and categorical variables, such as immunoreactivity and clinicopathological features. An association between categorical variables was analyzed using the χ2 test, a *p*-value ≤ 0.05 was considered significant. The median and interquartile range (IQR) were used to assess the degree of data dispersion around the centrality measure. The difference between the upper and lower quartiles was determined for the interquartile range. Heat maps were used to show hierarchical groupings of the gene expression profile of the samples, using the Z-score metric. The Kaplan-Meier estimator and the log-rank test were used to estimate the survival probability of the high and low expression groups defined by the cut-off points obtained by the Area-Under-the-Curve (AUC) for the *TTLL12*, *CDC16*, *UBE2T* and *MZT2B*. A 95% confidence level was considered and *p* <0.001. All statistics associated with clinical samples were performed using R (https://www.r-project.org).

### Data availability

The gene expression data used to support the findings of this study have been deposited in the Gene Expression Omnibus (GEO) repository under the access number GSE81265 (https://www.ncbi.nlm.nih.gov/geo/query/acc.cgi?acc=GSE 81265).

## Supplementary Material

Supplementary Table 1

Supplementary Table 2

Supplementary Table 3

Supplementary Table 4

Supplementary Table 5

Supplementary Table 6
