# Supplementary material for: Downregulated genes by silencing MYC pathway identified with RNA-SEQ analysis as potential prognostic biomarkers in gastric adenocarcinoma
Source: Aging (Albany NY). 2020 Dec 22;12(24):24651–70. doi: 10.18632/aging.202260 (PMC7803532; doi:10.18632/aging.202260)
Supplement: Supplementary Table 5 [file aging-12-202260-s005.docx]

**Supplementary Table 5. Results of MYC silencing on gene and protein expression in ACP02 and ACP03.**

| **Variables** | ***n*, 213 (%)** | | **TTLL12**  **immunoreactivity** | | ***TTLL12* mRNA** | | **TTLL12 protein** | | **CDKN3**  **immunoreactivity** | | ***CDKN3* mRNA** | | | **CDKN3 protein** | | | **CDC16**  **immunoreactivity** | | | ***CDC16* mRNA** | | | **CDC16 protein** | |
| --- | --- | --- | --- | --- | --- | --- | --- | --- | --- | --- | --- | --- | --- | --- | --- | --- | --- | --- | --- | --- | --- | --- | --- | --- |
|  |  |  | **N (%) of**  **positives**  **cases** | ***p*-value^a^** | **RQ (median ± IQR)** | ***p*-value^b^** | **Ratio T/N**  **(median ± IQR)** | ***p*-value^b^** | **N (%) of**  **positives**  **cases** | ***p*-value^a^** | **RQ (median ± IQR)** | ***p*-value^b^** | **Ratio T/N**  **(median ± IQR)** | | ***p*-value^b^** | **N (%) of**  **positives**  **cases** | | ***p*-value^a^** | **RQ (median ± IQR)** | | ***p*-value^b^** | **Ratio T/N**  **(median ± IQR)** | | ***p*-value^b^** |
| **Gender** | **Female** | **80 (37.6)** | 36 (33.0) | 0.477 | 1.40 ± 0.57 | 0.349 | 1.26 ± 0.63 | 0.588 | 79 (38.0) | < 0.001* | 1.48 ± 0.28 | 0.969 | 1.25 ± 0.22 | | 0.386 | 48 (42.5) | | 0.180 | 1.67 ± 0.58 | | 0.117 | 1.48 ± 0.62 | | 0.042 |
|  | **Male** | **133 (62.4)** | 73 (67.0) |  | 1.43 ± 0.76 |  | 1.44 ± 0.67 |  | 129 (62.0) |  | 1.48 ± 0.28 |  | 1.23 ± 0.25 | |  | 65 (57.5) | |  | 1.61 ± 0.50 | |  | 1.40 ± 0.52 | |  |
| **Age (years)** | **< 50 years** | **55 (25.8)** | 32 (29.4) | 0.564 | 1.51 ± 0.73 | 0.316 | 1.47 ± 0.59 | 0.359 | 53 (25.5) | < 0.001* | 1.36 ± 0.22 | 0.001 | 1.21 ± 0.26 | | 0.136 | 22 (19.5) | | 0.045 | 1.49 ± 0.47 | | 0.005 | 1.29 ± 0.46 | | 0.007 |
|  | **≥ 50 years** | **158 (74.2)** | 77 (70.6) |  | 1.41 ± 0.66 |  | 1.26 ± 0.69 |  | 155 (74.5) |  | 1.49 ± 0.27 |  | 1.24 ± 0.25 | |  | 91 (80.5) | |  | 1.68 ± 0.55 | |  | 1.47 ± 0.64 | |  |
| **Tumor location** | **Cardia** | **75 (35.2)** | 46 (42.2) | 0.100 | 1.50 ± 0.65 | 0.138 | 1.45 ± 0.55 | 0.053 | 74 (35.6) | < 0.001* | 1.46 ± 0.22 | 0.052 | 1.22 ± 0.26 | | 0.892 | 35 (31.0) | | 0.808 | 1.51 ± 0.65 | | 0.116 | 1.44 ± 0.56 | | 0.847 |
|  | **Non-cardia** | **138 (64.8)** | 63 (57.8) |  | 1.40 ± 0.64 |  | 1.22 ± 0.71 |  | 134 (64.4) |  | 1.49 ± 0.27 |  | 1.23 ± 0.23 | |  | 78 (69.0) | |  | 1.68 ± 0.50 | |  | 1.43 ± 0.62 | |  |
| **Histological type** | **Diffuse** | **103 (48.4)** | 93 (85.3) | < 0.001* | 1.78 ± 0.47 | < 0.001* | 1.70 ± 0.36 | < 0.001* | 100 (48.1) | 0.579 | 1.48 ± 0.27 | 0.123 | 1.22 ± 0.24 | | 0.241 | 45 (39.8) | | 0.020* | 1.55 ± 0.55 | | 0.062 | 1.37 ± 0.50 | | 0.015* |
|  | **Intestinal** | **110 (51.6)** | 16 (14.7) |  | 1.11 ± 0.33 |  | 1.08 ± 0.25 |  | 108 (51.9) |  | 1.49 ± 0.28 |  | 1.25 ± 0.23 | |  | 68 (60.2) | |  | 1.66 ± 0.51 | |  | 1.50 ± 0.63 | |  |
| **Stage** | **Early** | **20 (9.3)** | 15 (13.8) | 0.128 | 1.51 ± 0.57 | < 0.001* | 1.65 ± 0.47 | 0.053 | 19 (9.1) | < 0.001* | 1.08 ± 0.33 | < 0.001* | 0.99 ± 0.18 | | < 0.001* | 0 (0.0) | | < 0.001* | 1.20 ± 0.21 | | < 0.001* | 1.11 ± 0.18 | | < 0.001* |
|  | **Advanced** | **193 (89.4)** | 94 (86.2) |  | 1.41 ± 0.68 |  | 1.31 ± 0.66 |  | 189 (90.9) |  | 1.49 ± 0.27 |  | 1.25 ± 0.23 | |  | 113 (100.0) | |  | 1.68 ± 0.54 | |  | 1.48 ± 0.61 | |  |
| **Tumor invasion** | **T1/T2** | **68 (31.9)** | 53 (48.6) | 0.003* | 1.67 ± 0.74 | < 0.001* | 1.69 ± 0.61 | < 0.001* | 67 (32.2) | < 0.001* | 1.30 ± 0.16 | < 0.001* | 1.14 ± 0.20 | | < 0.001* | 10 (8.8) | | < 0.001* | 1.41 ± 0.39 | | < 0.001* | 1.27 ± 0.29 | | < 0.001* |
|  | **T3/T4** | **145 (68.1)** | 56 (51.4) |  | 1.38 ± 0.60 |  | 1.18 ± 0.60 |  | 141 (67.8) |  | 1.57 ± 0.12 |  | 1.28 ± 0.22 | |  | 103 (91.2) | |  | 1.73 ± 0.57 | |  | 1.56 ± 0.65 | |  |
| **Lymph node metastasis** | **Absent** | **23 (10.8)** | 17 (15.6) | 0.356 | 1.65 ± 0.38 | 0.177 | 1.45 ± 0.33 | 0.744 | 23 (11.1) | < 0.001* | 1.31 ± 0.20 | 0.004* | 1.13 ± 0.20 | | 0.033* | 0 (0.0) | | 0.003* | 1.33 ± 0.22 | | < 0.001* | 1.13 ± 0.22 | | < 0.001* |
|  | **Present** | **190 (89.2)** | 92 (84.4) |  | 1.40 ± 0.67 |  | 1.30 ± 0.69 |  | 185 (88.9) |  | 1.49 ± 0.27 |  | 1.25 ± 0.24 | |  | 113 (100.0) | |  | 1.68 ± 0.55 | |  | 1.49 ± 0.62 | |  |
| **Distant metastasis** | **Absent (M_0_)** | **108 (50.7)** | 52 (47.7) | 0.019* | 1.41 ± 0.64 | 0.538 | 1.29 ± 0.64 | 0.021* | 107 (51.4) | 0.677 | 1.47 ± 0.24 | 0.002* | 1.22 ± 0.23 | | 0.453 | 35 (31.0) | | 0.660 | 1.48 ± 0.33 | | < 0.001* | 1.26 ± 0.32 | | < 0.001* |
|  | **Present (M_1_)** | **105 (49.3)** | 57 (52.3) |  | 1.45 ± 0.74 |  | 1.52 ± 0.76 |  | 101 (48.6) |  | 1.56 ± 0.27 |  | 1.24 ± 0.24 | |  | 78 (69.0) | |  | 1.84 ± 0.52 | |  | 1.77 ± 0.68 | |  |
| **Survival 5 year** | **Negative** | **148 (69.5)** | 73 (67.0) | 0.182 | 1.40 ± 0.59 | 0.003* | 1.28 ± 0.63 | 0.194 | 144 (69.2) | < 0.001* | 1.50 ± 0.27 | < 0.001* | 1.28 ± 0.22 | | < 0.001* | 92 (81.4) | | < 0.001* | 1.70 ± 0.54 | | < 0.001* | 1.49 ± 0.63 | | 0.001* |
|  | **Positive** | **65 (30.5)** | 36 (33.0) |  | 1.59 ± 0.81 |  | 1.55 ± 0.72 |  | 64 (30.8) |  | 1.36 ± 0.22 |  | 1.13 ± 0.19 | |  | 21 (18.6) | |  | 1.44 ± 0.43 | |  | 1.29 ± 0.43 | |  |
| ***H. pylori* infection** | **Negative** | **23 (10.8)** | 13 (11.9) | 0.172 | 1.52 ± 0.50 | 0.364 | 1.50 ± 0.67 | 0.145 | 22 (10.6) | < 0.001* | 1.48 ± 0.27 | 0.769 | 1.22 ± 0.27 | | 0.711 | 12 (10.6) | | 0.724 | 1.51 ± 0.58 | | 0.168 | 1.45 ± 0.76 | | 0.437 |
|  | **Positive** | **190 (89.2)** | 96 (88.1) |  | 1.41 ± 0.68 |  | 1.32 ± 0.65 |  | 186 (89.4) |  | 1.48 ± 0.28 |  | 1.23 ± 0.24 | |  | 101 (89.4) | |  | 1.64 ± 0.51 | |  | 1.43 ± 0.58 | |  |
| ***H. pylori* CagA** | **Negative** | **73 (34.3)** | 43 (39.4) | 0.409 | 1.49 ± 0.68 | 0.228 | 1.55 ± 0.70 | 0.890 | 72 (34.6) | < 0.001* | 1.48 ± 0.27 | 0.464 | 1.23 ± 0.24 | | 0.473 | 37 (32.7) | | 0.733 | 1.55 ± 0.45 | | 0.097 | 1.45 ± 0.45 | | 0.738 |
|  | **Positive** | **140 (65.7)** | 66 (60.6) |  | 1.40 ± 0.67 |  | 1.24 ± 0.63 |  | 136 (65.4) |  | 1.49 ± 0.28 |  | 1.25 ± 0.24 | |  | 76 (67.3) | |  | 1.68 ± 0.57 | |  | 1.42 ± 0.62 | |  |
| **EBV** | **Negative** | **178 (83.6)** | 92 (84.4) | 0.880 | 1.42 ± 0.65 | 0.976 | 1.39 ± 0.62 | 0.425 | 173 (83.2) | < 0.001* | 1.48 ± 0.28 | 0.688 | 1.23 ± 0.25 | | 0.927 | 91 (80.5) | | 0.940 | 1.60 ± 0.49 | | 0.316 | 1.42 ± 0.53 | | 0.127 |
|  | **Positive** | **35 (16.4)** | 17 (15.6) |  | 1.44 ± 0.73 |  | 1.19 ± 0.79 |  | 35 (16.8) |  | 1.49 ± 0.27 |  | 1.22 ± 0.22 | |  | 22 (19.5) | |  | 1.69 ± 0.70 | |  | 1.60 ± 0.82 | |  |

**^a^***p* value by χ2 test; **^b^***p* value by Mann-Whitney test. **p*<0.05, significantly different between groups. n: number of samples; RQ: relative quantification, in which the matched non-neoplastic sample was designated as a calibrator from each neoplastic samples; T/N: the ratio of protein expression between neoplastic and matched non-neoplastic samples; IQR: interquartile range; CagA gene: Virulence factor cytotoxicity-associated gene A; EBV: Epstein-Barr virus. The patients' survival time was determined by the time interval between the date of surgery and the date of death.
