# Supplementary material for: Downregulated genes by silencing MYC pathway identified with RNA-SEQ analysis as potential prognostic biomarkers in gastric adenocarcinoma
Source: Aging (Albany NY). 2020 Dec 22;12(24):24651–70. doi: 10.18632/aging.202260 (PMC7803532; doi:10.18632/aging.202260)
Supplement: Supplementary Table 6 [file aging-12-202260-s006.docx]

**Supplementary Table 6. GSEA analysis of the DEGs in ACP02 and ACP03.**

| **Variables** | ***n*, 213 (%)** | | **PTPRA**  **immunoreactivity** | | ***PTPRA* mRNA** | | **PTPRA protein** | | **MZT2B**  **immunoreactivity** | | ***MZT2B* mRNA** | | **MZT2B protein** | | **UBE2T**  **immunoreactivity** | | | ***UBE2T* mRNA** | | | **UBE2T protein** | |
| --- | --- | --- | --- | --- | --- | --- | --- | --- | --- | --- | --- | --- | --- | --- | --- | --- | --- | --- | --- | --- | --- | --- |
|  |  |  | **N (%) of**  **positives**  **cases** | ***p*-value^a^** | **RQ (median ± IQR)** | ***p*-value^b^** | **Ratio T/N**  **(median ± IQR)** | ***p*-value^b^** | **N (%) of**  **positives**  **cases** | ***p*-value^a^** | **RQ (median ± IQR)** | ***p*-value^b^** | **Ratio T/N**  **(median ± IQR)** | ***p*-value^b^** | **N (%) of**  **positives**  **cases** | ***p*-value^a^** | **RQ (median ± IQR)** | | ***p*-value^b^** | **Ratio T/N**  **(median ± IQR)** | | ***p*-value^b^** |
| **Gender** | **Female** | **80 (37.6)** | 78 (37.9) | < 0.001* | 0.95 ± 0.39 | 0.184 | 0.94 ± 0.11 | 0.223 | 25 (47.2) | 0.680 | 1.30 ± 0.55 | 0.290 | 1.24 ± 0.60 | 0.061 | 80 (37.6) | < 0.001* | 1.64 ± 0.61 | | 0.189 | 1.51 ± 0.40 | | 0.077 |
|  | **Male** | **133 (62.4)** | 128 (62.1) |  | 0.94 ± 0.07 |  | 0.93 ± 0.06 |  | 28 (52.8) |  | 1.19 ± 0.44 |  | 1.13 ± 0.64 |  | 133 (62.4) |  | 1.56 ± 0.53 | |  | 1.42 ± 0.40 | |  |
| **Age (years)** | **< 50 years** | **55 (25.8)** | 54 (26.2) | 0.469 | 0.95 ± 0.65 | 0.038 | 0.93 ± 0.63 | 0.027 | 5 (9.4) | < 0.001* | 1.08 ± 0.47 | 0.031 | 0.99 ± 0.48 | 0.001 | 55 (25.8) | < 0.001* | 1.56 ± 0.62 | | 0.353 | 1.41 ± 0.38 | | 0.542 |
|  | **≥ 50 years** | **158 (74.2)** | 152 (73.8) |  | 0.94 ± 0.07 |  | 0.93 ± 0.05 |  | 48 (90.6) |  | 1.24 ± 0.49 |  | 1.25 ± 0.60 |  | 158 (74.2) |  | 1.59 ± 0.51 | |  | 1.47 ± 0.42 | |  |
| **Tumor location** | **Cardia** | **75 (35.2)** | 70 (34.0) | < 0.001* | 0.94 ± 0.47 | 0.413 | 0.93 ± 0.29 | 0.968 | 12 (22.6) | < 0.001* | 1.18 ± 0.42 | 0.434 | 1.09 ± 0.55 | 0.162 | 75 (35.2) | < 0.001* | 1.56 ± 0.61 | | 0.821 | 1.49 ± 0.42 | | 0.841 |
|  | **Non-cardia** | **138 (64.8)** | 136 (66.0) |  | 0.94 ± 0.07 |  | 0.93 ± 0.06 |  | 41 (77.4) |  | 1.24 ± 0.50 |  | 1.23 ± 0.68 |  | 138 (64.8) |  | 1.58 ± 0.52 | |  | 1.45 ± 0.40 | |  |
| **Histological type** | **Diffuse** | **103 (48.4)** | 101 (49.0) | 0.780 | 0.95 ± 0.59 | 0.138 | 0.93 ± 0.29 | 0.294 | 3 (5.7) | < 0.001* | 1.01 ± 0.25 | < 0.001* | 0.87 ± 0.35 | < 0.001* | 103 (48.4) | 0.631 | 1.61 ± 0.61 | | 0.322 | 1.42 ± 0.46 | | 0.410 |
|  | **Intestinal** | **110 (51.6)** | 105 (51.0) |  | 0.94 ± 0.06 |  | 0.93 ± 0.05 |  | 50 (94.3) |  | 1.43 ± 0.51 |  | 1.52 ± 0.45 |  | 110 (51.6) |  | 1.56 ± 0.46 | |  | 1.49 ± 0.39 | |  |
| **Stage** | **Early** | **20 (9.3)** | 20 (9.7) | < 0.001* | 1.66 ± 0.42 | < 0.001* | 1.74 ± 0.28 | < 0.001* | 0 (0.0) | < 0.001* | 0.93 ± 0.42 | 0.002* | 0.77 ± 0.27 | < 0.001* | 20 (9.4) | < 0.001* | 1.26 ± 0.23 | | < 0.001* | 1.29 ± 0.21 | | 0.001* |
|  | **Advanced** | **193 (89.4)** | 186 (90.3) |  | 0.94 ± 0.06 |  | 0.93 ± 0.04 |  | 53 (100.0) |  | 1.22 ± 0.46 |  | 1.24 ± 0.60 |  | 193 (90.6) |  | 1.63 ± 0.50 | |  | 1.48 ± 0.43 | |  |
| **Tumor invasion** | **T1/T2** | **68 (31.9)** | 68 (33.0) | 0.650 | 1.54 ± 0.66 | < 0.001* | 1.51 ± 0.78 | < 0.001* | 3 (5.7) | < 0.001* | 1.03 ± 0.33 | < 0.001* | 1.05 ± 0.50 | < 0.001* | 68 (31.9) | < 0.001* | 1.29 ± 0.41 | | < 0.001* | 1.29 ± 0.24 | | < 0.001* |
|  | **T3/T4** | **145 (68.1)** | 138 (67.0) |  | 0.93 ± 0.04 |  | 0.92 ± 0.02 |  | 50 (94.3) |  | 1.29 ± 0.45 |  | 1.29 ± 0.64 |  | 145 (68.1) |  | 1.70 ± 0.46 | |  | 1.52 ± 0.42 | |  |
| **Lymph node metastasis** | **Absent** | **23 (10.8)** | 23 (11.2) | 0.486 | 1.53 ± 0.73 | 0.003* | 1.54 ± 0.82 | 0.002* | 1 (1.9) | < 0.001* | 0.99 ± 0.41 | 0.001* | 0.84 ± 0.38 | 0.001* | 23 (10.8) | < 0.001* | 1.32 ± 0.54 | | 0.003* | 1.29 ± 0.18 | | < 0.001* |
|  | **Present** | **190 (89.2)** | 183 (88.8) |  | 0.94 ± 0.07 |  | 0.93 ± 0.05 |  | 52 (98.1) |  | 1.24 ± 0.44 |  | 1.23 ± 0.61 |  | 190 (89.2) |  | 1.60 ± 0.52 | |  | 1.48 ± 0.44 | |  |
| **Distant metastasis** | **Absent** | **108 (50.7)** | 108 (52.4) | 0.064 | 0.94 ± 0.62 | 0.343 | 0.93 ± 0.62 | 0.378 | 13 (24.5) | < 0.001* | 1.17 ± 0.41 | < 0.001* | 1.08 ± 0.55 | 0.001* | 114 (53.5) | 0.304 | 1.51 ± 0.52 | | 0.001* | 1.38 ± 0.33 | | < 0.001* |
|  | **Present** | **105 (49.3)** | 98 (47.6) |  | 0.95 ± 0.05 |  | 0.93 ± 0.04 |  | 40 (75.5) |  | 1.26 ± 0.74 |  | 1.38 ± 0.80 |  | 99 (46.5) |  | 1.69 ± 0.51 | |  | 1.50 ± 0.51 | |  |
| **Survival 5 year** | **Negative** | **148 (69.5)** | 143 (69.4) | < 0.001* | 0.94 ± 0.06 | 0.005* | 0.93 ± 0.04 | 0.047* | 42 (79.2) | < 0.001* | 1.25 ± 0.46 | 0.540 | 1.23 ± 0.59 | 0.030* | 148 (69.5) | < 0.001* | 1.63 ± 0.49 | | 0.004* | 1.50 ± 0.47 | | 0.001* |
|  | **Positive** | **65 (30.5)** | 63 (30.6) |  | 0.97 ± 0.65 |  | 0.94 ± 0.64 |  | 10 (18.9) |  | 1.12 ± 0.49 |  | 1.07 ± 0.57 |  | 65 (30.5) |  | 1.45 ± 0.45 | |  | 1.34 ± 0.24 | |  |
| ***H. pylori* infection** | **Negative** | **23 (10.8)** | 22 (10.7) | < 0.001* | 0.95 ± 0.59 | 0.559 | 0.93 ± 0.24 | 0.819 | 2 (3.8) | < 0.001* | 0.96 ± 0.37 | 0.002* | 0.99 ± 0.54 | 0.034* | 23 (10.8) | < 0.001* | 1.48 ± 0.54 | | 0.126 | 1.30 ± 0.43 | | 0.314 |
|  | **Positive** | **190 (89.2)** | 184 (89.3) |  | 0.94 ± 0.08 |  | 0.93 ± 0.06 |  | 51 (96.2) |  | 1.24 ± 0.46 |  | 1.18 ± 0.63 |  | 190 (89.2) |  | 1.60 ± 0.54 | |  | 1.45 ± 0.40 | |  |
| ***H. pylori* CagA** | **Negative** | **73 (34.3)** | 72 (35.0) | < 0.001* | 0.95 ± 0.55 | 0.316 | 0.93 ± 0.23 | 0.514 | 11 (20.8) | < 0.001* | 1.14 ± 0.46 | 0.500 | 1.11 ± 0.51 | 0.084 | 73 (34.3) | < 0.001* | 1.50 ± 0.50 | | 0.034* | 1.45 ± 0.39 | | 0.848 |
|  | **Positive** | **140 (65.7)** | 134 (65.0) |  | 0.94 ± 0.07 |  | 0.93 ± 0.05 |  | 42 (79.2) |  | 1.25 ± 0.50 |  | 1.18 ± 0.71 |  | 140 (65.7) |  | 1.63 ± 0.52 | |  | 1.45 ± 0.45 | |  |
| **EBV** | **Negative** | **178 (83.6)** | 171 (83.0) | < 0.001* | 0.94 ± 0.25 | 0.852 | 0.93 ± 0.08 | 0.980 | 41 (77.4) | < 0.001* | 1.20 ± 0.44 | 0.345 | 1.14 ± 0.67 | 0.094 | 178 (83.6) | < 0.001* | 1.56 ± 0.55 | | 0.339 | 1.45 ± 0.40 | | 0.462 |
|  | **Positive** | **35 (16.4)** | 35 (17.0) |  | 0.94 ± 0.07 |  | 0.93 ± 0.05 |  | 12 (22.6) |  | 1.26 ± 0.55 |  | 1.35 ± 0.79 |  | 35 (16.4) |  | 1.63 ± 0.47 | |  | 1.49 ± 0.39 | |  |
